# A Review of Nutritional Factors in Hypertension Management

**DOI:** 10.1155/2013/698940

**Published:** 2013-04-10

**Authors:** Ha Nguyen, Olaide A. Odelola, Janani Rangaswami, Aman Amanullah

**Affiliations:** ^1^Department of Medicine, Albert Einstein Medical Center, Philadelphia, PA 19141, USA; ^2^Noninvasive Cardiology, Albert Einstein Medical Center, Clinical Professor of Medicine, Jefferson Medical College of Thomas Jefferson University, 5501 Old York Road, HB-3, Philadelphia, PA 19141, USA

## Abstract

Hypertension is a major health problem worldwide. Its attendant morbidity and mortality complications have a great impact on patient's quality of life and survival. Optimizing blood pressure control has been shown to improve overall health outcomes. In addition to pharmacological therapies, nonpharmacological approach such as dietary modification plays an important role in controlling blood pressure. Many dietary components such as sodium, potassium, calcium, and magnesium have been studied substantially in the past decades. While some of these nutrients have clear evidence for their recommendation, some remain controversial and are still of ongoing study. Dietary modification is often discussed with patients and can provide a great benefit in blood pressure regulation. As such, reviewing the current evidence will be very useful in guiding patients and their physician and/or dietician in decision making. In this review article of nutritional factors in hypertension management, we aim to examine the role of nutritional factors individually and as components of whole dietary patterns.

## 1. Introduction

In adults aged 18 years and older, hypertension is defined as a systolic blood pressure (SBP) equal or more than 140 mmHg and/or diastolic blood pressure (DBP) equal or more than 90 mmHg based on the mean of 2 or more properly measured seated blood pressure readings on each of 2 or more office visits by the seventh report of the Joint National Committee on Prevention, Detection, Evaluation, and Treatment of High Blood Pressure (JNC) [1]. According to the 2012 World Health Statistics report released by the World Health Organization (WHO), hypertension affects approximately 24.8% of the global population with the range from 19.7% to 35.5% in different regions [2]. It is one of the most common diseases that lead to office visits or hospitalizations and a major risk factor for stroke, congestive heart failure (CHF), myocardial infarction (MI), peripheral vascular disease, and overall mortality. Many of those with hypertension are undiagnosed, and of those detected, about two-thirds are suboptimally controlled [1]. Early treatment can improve blood pressure and its complications significantly [1]. Therapeutic options include diet and lifestyle changes (including weight loss, smoking cessation, and increased physical activity), antihypertensive drugs, and surgery in special situations.

Clinical and population-based studies show that several components of the diet such as sodium, potassium, calcium, magnesium, fiber, and fish oil affect blood pressure, and modification of these nutritional factors provide an important strategy to control blood pressure especially in the prehypertensive stage (SBP 120–139 mmHg and/or DBP 80–89 mmHg) or stage I hypertension (SBP 140–159 mmHg and/or DBP 90–99 mmHg). The role of these dietary factors singly or in combination in blood pressure regulation and to what extent each contributes has been a subject of research for many decades, and despite this, it remains controversial. Modifying one's diet can be a difficult and significant change in life, and many patients oppose this or fail despite several attempts. Given how frequently clinicians have to provide patients with this recommendation and the potential impact on the overall outcome in patient health, it is of great benefit to study the current evidence in nutritional approach to hypertension management. In this paper, we aim to review the role of each individual dietary factor as well as whole dietary patterns.

## 2. Sodium

The relationship between sodium intake and blood pressure changes has been a topic of discussion for decades. Hypertension is predominantly observed in societies with average sodium chloride intake >100 mmol/day and very rare in populations consuming <50 mmol/day [3, 4]. However, the link between salt and hypertension is still an ongoing debate. The concept of salt sensitivity versus salt resistance originated from studies demonstrating heterogeneous blood pressure responses to changes in sodium intake. These changes were observed both in hypertensive and normotensive subjects [5–8]. Till date, there is no uniformed definition of salt sensitivity due to great variations in published studies regarding study protocols, techniques, duration, and magnitude of sodium intake and blood pressure changes. However, the most commonly used method was introduced by Weinberger [9]. Based on mean arterial blood pressure response to sodium load by intravenous administration of 2 liters of normal saline and sodium depletion by a 10 mmol sodium diet and 3 doses of oral furosemide (40 mg each), the authors arbitrarily classified salt sensitivity as a decrease in mean arterial pressure ≥10 mmHg and salt resistance as a decrease ≤5 mmHg when comparing the 2 blood pressure measurements. 

As a brief review of physiology, sodium homeostasis is maintained by glomerular filtration and tubular reabsorption. Sixty-five to seventy-five percent of sodium reabsorption is mediated by neurohumoral hormone in the proximal (angiotensin II and norepinephrine) and collecting tubules (aldosterone, atrial natriuretic peptide), whereas 30–35% occurs in the loop of Henle and distal tubules and this is flow dependent. A parallel adaptation in renal tubular activity in response to alterations in glomerular filtration of sodium keeps little variation in urinary sodium excretion [10]. This explains a steady range of sodium level even in cases of advanced kidney failure. 

Salt sensitivity has been found to have a higher prevalence in certain populations: older age [11, 12], blacks [12], insulin resistance [13, 14], microalbuminuria [15], chronic kidney disease (CKD) [16], and low renin level [17, 18]. Recent animal and molecular studies have suggested the contribution of certain genetic polymorphisms to the development of salt sensitive hypertension via different mechanisms: affecting renal tubular Na+K+ATPase activity [19, 20], decreasing dopamine receptor function in renal proximal tubule [21], or altering endothelin receptor activity [22]. Salt sensitivity has been reported to be an independent prognostic factor for increased risk of left ventricular hypertrophy [23], cardiovascular events [24], and cumulative mortality [25], regardless of blood pressure.

Dietary sodium restriction is strongly advocated as a lifestyle behavioral change for prevention and treatment of hypertension and consequently cardiovascular morbidity and mortality by several professional organizations [1, 26–28]. Despite an abundance of studies on its efficacy, results are conflicting and naturally provoke questions on the benefits of continuing to advocate this intervention to patients [29, 30].

Evidence from several clinical studies shows that a reduction in sodium intake leads to modest to large reduction in blood pressure in normotensive and hypertensive participants [31–36] as well as decreased risk of cardiovascular events [37–39].

The INTERSALT study, a population-based study across 52 centers from 32 countries, examined the relationship between sodium excretion and blood pressure among over 10,000 participants. Sodium excretion was highly variable (0.2–242 mmol/24 hour (hr)), with 4 centers having very low levels (0.2–51.3 mmol/24 hr). A significant positive association between sodium excretion and blood pressure in individuals was reported overall. However, with exclusion of data from the 4 centers with the lowest excretion, no relation could be found [4].

The Dietary Approaches to Stop Hypertension (DASH)-sodium trial randomized subjects to either the DASH diet (rich in fruits, vegetables, and low-fat dairy and reduced in saturated and total fat) or a control diet (typical United States (US) diet which is high in fat and low in fruits, vegetables, and dairy products) with participants consuming a graded sodium intake (high, intermediate, low) within each group during the study period. The study demonstrated significant dose-response decreases in SBP and DBP overall and blunting of the age-related increase in blood pressure with sodium restriction. The DASH diet and sodium restriction each lowered blood pressure substantially, but the effect was greater when combined [35, 36].

Investigators on a prospective followup study of participants in the trials of hypertension prevention phase I and II for 10–15 years found a 30% and 20% risk reduction of cardiovascular disease (MI, stroke and coronary revascularization) and cardiovascular death, respectively, but the reduction in death was not statistically significant [37].

In contrast, several studies demonstrate harmful effects of lower salt intake on important clinical outcomes including MI [40], all-cause and cardiovascular mortality [41, 42], sympathetic hormones [43, 44], fasting plasma glucose and insulin levels [44, 45], cholesterol [43, 44, 46], and in addition, higher CHF readmissions and brain natriuretic peptide levels [47, 48]. Higher mortality [49, 50] and development of end stage renal disease (ESRD) [49] have also been reported with lower sodium intake among diabetics. 

All together, despite several years and high magnitude of population-based studies and clinical research, the evidence supporting the recommendation of dietary sodium restriction should be interpreted with caution.

Given that salt restriction is not a “one size fits all” approach, differentiating individuals with salt sensitive and salt resistant hypertension would be beneficial. However, subjecting all hypertensive patients to salt sensitivity testing as in research protocols seems inconvenient and unfeasible. Thus, the question appears to be how physicians can clinically determine salt sensitivity. To address this query, a couple of studies have suggested ambulatory, however, indirect methods in evaluating salt responsiveness. De la Sierra and coinvestigators reported that patient's performance on ambulatory blood pressure monitoring could be a useful tool to assess salt sensitivity [51]. In their study, salt sensitive patients exhibited a nondipper profile on both low salt and high salt diets, while salt resistant subjects with high salt intake had increased blood pressure during sleep with no significant changes in the 24-hr blood pressure. Galletti et al. found that in salt sensitive patients, there was a strong correlation between 24-hr urinary sodium excretion and blood pressure changes [52]. In another study, involving 89 Caribbean Hispanic hypertensive patients, plasma renin level was used to evaluate patient's response to antihypertensive therapy [53]. Sixty-two percent of the patients had low renin essential hypertension and responded preferentially to monotherapy with hydrochlorothiazide or calcium channel blockers. A phase 4 randomized study testing Fludrocortisone administration in identifying salt sensitivity is currently underway [54]. These methods need to be validated by larger studies before being considered for routine tests in evaluating and/or guiding treatment in hypertensive patients. 

Currently, based on available evidence, the WHO strongly recommends the restriction of daily sodium intake to less than 2000 mg (or 5000 mg salt) [55], while the AHA advises lower than 1500 mg sodium (or 3800 mg salt) per day [28].

## 3. Potassium

The association of potassium and blood pressure has been described in many studies. Potassium intake was found to be inversely related to both DBP and SBP in a population-based study including 685 men and women who were predominantly Caucasian in Southern California, United States of America (USA) [56]. Similar result was illustrated in the Rotterdam Study [57], a big population-based study in which 3239 participants older than 55 years old were included. Patients with an increase in potassium intake of 1000 mg/day had a 0.9 mmHg lower SBP and a 0.8 mmHg lower DBP. In a study by Krishna and Kapoor, potassium depletion was shown to be associated with a decrease in sodium excretion, plasma renin activity, and plasma aldosterone concentrations and an increase of 7 mmHg in SBP and 6 mmHg in DBP [58].

Several interventional studies have shown the positive effects of potassium supplementation on blood pressure reduction. Cappuccio and MacGregor reviewed 19 clinical trials in which oral potassium supplements significantly lowered SBP (mean of −5.9 mmHg, 95% confidence internal (CI), −6.6 to −5.2 mmHg) and DBP (mean of −3.4 mmHg, 95% CI, −4.0 to 2.8 mmHg) [59]. A meta-analysis consisting of 27 potassium trials in adults with a minimum of 2 weeks duration also demonstrated a change in blood pressure with increased potassium intake: a mean of −2.42 mmHg (95% CI, −3.75 to −1.08 mmHg) in SBP and −1.57 mmHg (95% CI, −2.65 to −0.50 mmHg) in DBP pressure [60]. Dickinson and colleagues used stricter inclusion criteria and only selected 5 randomized controlled trials for their meta-analysis published in the Cochrane Database in 2006. The authors did not find a statistical significant effect of potassium supplementation on blood pressure. However, the argument of small number of subjects, short duration of followup, and substantial heterogeneity of these trials was discussed as a possible explanation for the finding of this meta-analysis [61].

The benefit of potassium intake on blood pressure reduction appears to be greater in patients with hypertension [59, 60], longer duration of supplementation [59], and concurrent high intake of sodium [62].

The magnitude of blood pressure reduction seems small in number but might be translated into a benefit in mortality from complications of hypertension. In a study of 6 different populations, the relative increase in 25-year mortality risk due to coronary heart disease was 1.17 (95% CI, 1.14 to 1.20) per 10 mmHg increase in SBP and 1.13 (95% CI, 1.10 to 1.15) per 5 mmHg increase in DBP. After adjustment for within-subject variability in blood pressure, this relative risk was 1.28 [63]. A 5 mmHg decrease in DBP was reported to be associated with less than one-third of strokes [64, 65].

It is worth mentioning that some protective effects of high potassium intake on cardiovascular health might be independent of blood pressure. An analysis of sodium and potassium intake and mortality among US adults based on data from the third National Health and Nutrition Examination Survey (NHANES) showed that higher sodium intake was associated with increased all-cause mortality, while higher potassium intake seemed to be associated with lower mortality. Of note, this finding was independent of sex, age, hypertension, physical activity, and body mass index (BMI) [66]. In a 12-year prospective study, a 10 mmol increase in daily potassium intake was associated with a 40% reduction in the risk of stroke-associated mortality. This protective effect of potassium did not differ by other dietary variables and known cardiovascular risk factors (age, sex, blood pressure, blood cholesterol level, obesity, fasting blood glucose level, and cigarette smoking) [67].

The antihypertensive effect of potassium supplementation might be from various mechanisms: (1) natriuresis by inhibiting sodium reabsorption in the proximal renal tubules [68] and suppressing renin secretion [69], (2) normalization of the plasma level of digitalis like substance [70], (3) increased urinary volume excretion [70], (4) smooth muscle relaxation [71, 72] by increasing nitric oxide production [73] and/or by stimulating the rectifier K(+) channels resulting in potential membrane hyperpolarization and subsequently vasodilation [74], (5) suppression of free radical formation [75], and (6) protection against vascular injury in salt sensitive hypertension [76]. Which one of these mechanisms plays a predominant role in the reduction of blood pressure and/or cardiovascular mortality is not quite clear at this time.

Based on available data, the Institute of Medicine has recommended a potassium intake of 4700 mg (120 mmol) a day as adequate intake for all adults [77]. A similar amount of daily potassium consumption was also suggested by the American Heart Association (AHA) in 2006 to achieve the potential benefit of blood pressure reduction [28]. The 2003 WHO/International Society of Hypertension statement on management of hypertension supported an increased dietary potassium intake although a threshold was not specified [78]. However, the suggested daily intake of potassium might be lower in patients who are prone to developing hyperkalemia such as those with impaired renal excretion of potassium from CKD, CHF, adrenal insufficiency, and medications use (angiotensin converting enzyme (ACE) inhibitors, angiotensin receptor blockers (ARB), potassium-sparing diuretics, trimethoprim, cyclosporine, heparin, etc.) These patients need to be monitored frequently.

In reality, the average intake of dietary potassium in many countries is relatively lower than recommended. As in a report from the third NHANES 1988–1994 study, estimated mean potassium intakes of adults in the USA ranged from 2900 to 3300 mg in men and 2200 to 2400 mg in women [28]. The NHANES data from 2003 to 2008, the time span during which the above recommendations were issued, revealed that less than 2% of adults and approximately 5% of men in the USA met the recommendations for potassium intake (i.e., at least 4700 mg/day) [79]. In European countries, the average daily potassium intake in adults was reported to be below 4700 mg. The number varied from 3200 mg to 4000 mg/day in Finland [80] or from 2655 mg to 3371 mg/day in the United Kingdom [81]. In China, the mean intake of potassium was 1950 mg/day in the urban and 1830 mg/day in the rural diet [82].

Foods rich in potassium are vegetables, fruit, dairy products, nuts, and so forth. Natural source of potassium is preferable. Currently, pharmacological potassium supplementation is not recommended as a method to obtain the advised daily intake of potassium.

## 4. Calcium

The relationship between calcium intake and hypertension is a complex and difficult one to isolate largely because of the interaction with other nutrients in the diets and difficulty in reliably collecting calcium intake data and important unmeasured confounding variables.

Despite an abundance of studies on the effect of dietary or supplemental calcium on blood pressure, the evidence on its benefit is inconclusive and remains controversial. An inverse relationship between dietary calcium intake and blood pressure has been reported in many studies [83–89]. Similarly, supplementation with 1000 mg calcium/day has been demonstrated to lead to a decrease in blood pressure, although results were inconsistent and mainly among hypertensives [90–92]. In contrast, other studies report minimal to no effect of dietary calcium or supplementation on blood pressure [93–99]. In addition, contradictory results have been deduced from analysis of the same data as exemplified by the Nurses' Health Study [85, 94] and the NHANES [89, 100–103]. The differences in study results may be explained by a variety of factors including heterogeneity of study participants, flaws in study design, limitations in measurement of blood pressure and calcium intake, short duration of studies, different analytical methods, as well as collinearity with other dietary factors such as magnesium, fiber, protein, and potassium. 

Two meta-analyses assessed the relationship between dietary calcium supplementation and blood pressure. The authors found small reductions in SBP (1-2 mmHg), and results were even smaller and insignificant for DBP [104, 105].

Similar results were reported in another meta-analysis by van Mierlo et al., effect on SBP of −1.86 mmHg (95% CI, −2.91 to −0.81 mmHg) and DBP of −0.99 mmHg (95% CI, −1.61 to −0.37 mmHg), however, the impact on SBP was larger in people with relatively low calcium intake of ≤800 mg/day [106]. A Cochrane review published in 2006 examined the efficacy of oral calcium supplementation as a treatment for hypertension. Only 13 randomized controlled trials with a total of 485 participants with at least 8 weeks duration were included. A statistically significant reduction in SBP (−2.5 mmHg, 95% CI, −4.5 to −0.6 mmHg), but not DBP (−0.8 mmHg, 95% CI, −2.1 to 0.4 mmHg), was found. Many of the trials were of poor quality, and the authors concluded that there was insufficient evidence to recommend calcium supplementation as a treatment for hypertension [107].

Currently, the evidence on the benefit of calcium supplementation in prevention or treatment of hypertension is weak; therefore, there is no justification to increase the intake of calcium above the recommended dietary allowance of 1000–1300 mg/day based on age and gender. Foods rich in calcium are mainly dairy products (preferably low fat) such as milk, cheese, and yogurt.

Proposed mechanisms by which calcium intake regulates blood pressure include alteration in intracellular calcium which in turn affects vascular smooth muscle contraction [108], effect of calcium metabolism and regulatory hormones [109–111], increased natriuresis [112–114], and modulation of the function of the sympathetic nervous system [113]. Interestingly, among people consuming low levels of calcium in their diets, high salt intake is associated with higher blood pressure levels [115–117], and it is suggested that the hypertensive effect of a high sodium intake may be mitigated by increasing dietary calcium [109, 112, 118, 119]. Resnick also published extensively on the interlinking of the renin-aldosterone system, calcium regulation, and salt sensitivity in modulating blood pressure responses to salt loading, calcium supplementation, and calcium channel blockers [120–125]. He suggested that these models may provide a targeted approach to identifying and treating hypertensives with calcium supplementation or calcium channel blockers based on their serum renin level and salt sensitivity [126–128].

## 5. Magnesium

Magnesium deficiency has been found to result in increased blood pressure in single studies [129, 130]. However, a meta-analysis looking at 29 observational studies points to a negative correlation between dietary magnesium intake and blood pressure [131].

The evidence of a causal association between magnesium supplementation and blood pressure reduction was weak in a meta-analysis including 12 randomized trials with followup ranging from 8 to 26 weeks [132]. In another meta-analysis with 20 studies, although a dose-response pattern was found, magnesium intake only resulted in a small overall reduction in blood pressure, a mean of −0.6 mmHg (95% CI, −2.2 to 1.0 mmHg) for SBP and −0.8 mm Hg (95% CI, −1.9 to 0.4 mmHg) for DBP [133].

On the basis of these data, the relationship between magnesium and hypertension seems inconsistent and not convincing. Currently, magnesium supplementation is not recommended as a means of hypertensive treatment.

## 6. Alcohol

A standard drink in the USA is equal to 14 g (6 ounces (oz)) of pure alcohol. This amount is present in 12 oz of regular beer, 5 oz of wine (12% alcohol), 8 oz of malt liquor, and 1.5 oz of 80-proof distilled spirits [134]. This serving size can be varied in different countries. In Britain or Australia, a unit of alcohol contains 8 to 10 g [135], while in Japan, a drink contains 19.75 g of alcohol. In this paper, we refer “a drink” to the US serving size. Otherwise, the amount of alcohol will be specified.

Moderate alcohol intake, defined as a maximum of 2 alcoholic drinks/day in men and 1 alcoholic drink/day in women and lighter-weight persons, is supported by the AHA 2006 scientific statement of hypertension management [28]. In a prospective cohort study involving 11,711 men with preexisting hypertension, individuals who consumed a moderate amount of alcohol tended to have a decreased risk of MI. After adjustment for measurement error, BMI and dietary variables, the hazard ratio for participants with MI per 12.5 g/day increment of alcohol intake was 0.68 (95% CI, 0.46 to 1.00) [136].

On the other hand, heavy drinking, generally referred to as any amount of alcohol use above the moderate level, is associated with a higher risk of hypertension in a dose-dependent manner. This has been demonstrated in various populations: Japanese men [137], US women [138], and both men and women of different races [139]. The risk seems more pronounced in individuals with a smaller BMI [137]. In the INTERSALT study, compared to nondrinkers, men who drank 300–499 mL alcohol/week had higher SBP/DBP, on average 2.7/1.6 mmHg. Women who drank at least 300 mL/week had blood pressures higher by 3.9/3.1 mmHg than nondrinkers [140]. Alcohol reduction was associated with a fall in blood pressure [141]. Available data supports that moderate alcohol consumption should be recommended as one of the components of hypertensive therapy.

## 7. Fiber

Fiber is the indigestible portion from plant-based food. How exactly fiber might affect blood pressure is not entirely understood. An inverse association between blood pressure and fiber intake has been described. Among 30,681 predominantly white US male health professionals, 40–75 years old, those with a fiber intake of less than 12 g/day were at higher risk of developing hypertension compared to those taking more than 24 g/day, relative risk 1.57 (95% CI, 1.20 to 2.05) [88]. This relationship was independent of other nutrients including sodium, potassium, calcium, and magnesium. When the results of 24 randomized controlled trials were evaluated, fiber supplementation with an average dose of 11.5 g/day modestly reduced SBP by −1.13 mmHg (95% CI, −2.49 to 0.23 mmHg) and DBP by −1.26 mmHg (95% CI, −2.04 to −0.48 mmHg). It seemed to have greater effect in populations with hypertension and in those older than 40 years of age [142]. Similarly, a large randomized trial failed to show a significant decrease in blood pressure with a high fiber diet [143]. According to the 2006 scientific statement from the AHA, there is insufficient evidence to recommend increased dietary fiber intake alone for the reduction of blood pressure [28].

## 8. Omega-3 Polyunsaturated Fatty Acid (Fish Oil)

Daily dietary fish consumption as a part of weight reduction regimens has shown benefits in blood pressure fall [144]. A body of evidence demonstrated the association between concentrated Omega-3 polyunsaturated fatty acid extracted from fish, hence usually known as “fish oil”, and blood pressure reduction [145, 146]. This effect might have a dose limit, as it was reported that fish oil did not change mean blood pressure in the subjects who ate fish three or more times a week as part of their usual diet, or in those who had a baseline concentration of plasma phospholipid omega-3 fatty acids above 175.1 mg/L [146]. Of note, these studies employed large doses of Omega-3 polyunsaturated fatty acid (at least 3 g/day), which raises a concern of safety and side effects in view of long-term use. 

Mercury is a chemical element of which fish living in contaminated water can contain a high level in their body. Some cross-sectional studies have mentioned the possibility of increasing blood pressure caused by mercury. Would this go against the above findings of a positive correlation between daily fish meal and blood pressure reduction? Recently, two large prospective cohort studies on 6045 US men and women, do not support any effects of methylmercury on the risk of incident hypertension [147].

## 9. Garlic

Garlic is one of the most commonly used natural herbs. Its role in hypertension has also been explored by researchers. In patients with baseline elevated blood pressure, compared with placebo, garlic significantly reduced SBP by 16.3 mmHg (95% CI, 6.2 to 26.5 mmHg) and DBP by 9.3 mmHg (95% CI, 5.3 to 13.3 mmHg) [148]. The effect was repeatedly illustrated in another meta-analysis [149]. Despite the potential advantage, the problem with nonstandardization in product preparation, formulas, and study heterogeneity does not allow a conclusive message regarding garlic use in hypertensive patients. It might be beneficial. However, how much, how long, and which products to use are not clearly identified. 

## 10. Products with Potential Harms

### 10.1. Caffeine

Being the main ingredient in stimulant drinks, caffeine is found in coffee, tea, sodas, and many energy drinks. Acutely, caffeine can elevate blood pressure in nonhabitual caffeine users [150], whereas little to no effect was seen in habitual coffee drinkers [151, 152]. Chronic coffee drinkers who have hypertension might not need to change their habit based on the available data. Nevertheless, it can potentially be harmful for irregular coffee drinkers.

There is evidence that other ingredient than caffeine might be responsible for the stimulating effect of coffee on blood pressure. An increase in blood pressure was observed in decaffeinated coffee drinkers [153]. The use of caffeine alone did not exert an elevated blood pressure in healthy volunteers in a study [152].

### 10.2. Licorice

Licorice has been long used as a flavoring agent in chewing tobacco, candies, spices, and as a medical product in some gastrointestinal and upper respiratory disorders. It contains glycyrrhetinic acid which inhibits 11-beta-hydroxysteroid dehydrogenase enzyme type 2 isoform, allowing cortisol to bind to the mineralocorticoid receptors creating a status of mineralocorticoid excess and subsequently blood pressure elevation. The use of licorice can be potentially dangerous in hypertensive individuals [154].

## 11. Whole Dietary Pattern

### 11.1. DASH Diet

The DASH diet is a diet rich in fruits and vegetables (4-5 servings/day) and low-fat dairy products (2-3 servings/day) and includes whole grains, poultry, fish, and nuts. This diet is rich in potassium, magnesium, calcium, dietary fiber, and protein and has reduced fat (total and saturated) and cholesterol (<25%), red meat, sweets, and sugar-containing beverages. 

Two controlled clinical trials established its efficacy in lowering blood pressure [35, 155]. The initial DASH trial [155] enrolled 459 untreated participants with prehypertension and stage I hypertension and randomly assigned them to one of 3 groups (1) a control group which consumed a typical US diet which was low in fruits, vegetables, and dairy products and had a high fat content, (2) a diet rich in fruits and vegetables, or (3) the DASH diet sodium intake and body weight were held constant during the study period of eight weeks. Compared with the control group, blood pressure decreased by 5.5/3.0 mmHg and 2.8/1.1 mmHg in the DASH diet and fruits-and-vegetables diet, respectively. This reduction was higher among the subset of hypertensives at 11.4/5.5 mmHg compared with 3.5/2.1 mmHg for those without hypertension consuming the DASH diet. In addition, the reduction in blood pressure began within two weeks and was sustained for the next six weeks. 

The DASH-sodium trial [35] was a crossover trial in which 412 subjects were randomized to either the control diet or DASH diet and 3 levels of sodium intake (low: 1.2 g/day, intermediate: 2.3 g/day, and high: 3.5 g/day) within each diet arm for four weeks. Participants had SBP between 120 to 159 mmHg and DBP between 80 to 95 mmHg. Similar to the earlier study, the DASH diet significantly lowered blood pressure independent of sodium intake. With each diet, reducing the sodium intake significantly decreased blood pressure, and these effects persisted among those with and without hypertension, as well as across the different races and sex. The combination of the DASH diet and low sodium intake had the greatest impact, reducing SBP by 11.5/5.7 mmHg and 7.1/3.1 mmHg among hypertensives and those without hypertension, respectively, compared with the control diet and high sodium intake. The level of reduction among hypertensives is comparable to that obtained with drug monotherapy. 

African Americans with hypertension derive the most benefit from the blood pressure lowering effect of the DASH diet [156], however they may be less likely to adhere to the diet [157, 158]. Furthermore, the increase in blood pressure with aging can be reduced by adopting the DASH diet with sodium restriction [36, 159].

Other potential benefits of the DASH diet include reduction in cardiovascular morbidity [160, 161], mortality [162], CHF events [163, 164], and cardiovascular risks factors [165–167] as well as prevention of type 2 diabetes [168].

The DASH and low sodium diet has been endorsed by professional organizations including the AHA [169], the JNC [1], the American Association of Clinical Endocrinologists [170], and the Canadian Hypertension Education Program [27] for the prevention and treatment of hypertension.

Barriers to compliance with the DASH diet include cost, availability, accessibility, lack of information, and cultural dietary preferences. The US Department of Health and Human Services published a guidebook, *Your Guide to Lowering your Blood Pressure with DASH*, which is a great resource to guide patients in preparation of meals in accordance with the DASH diet [171].

The PREMIER trial studied 810 adults with SBP ranging from 120 to 159 mmHg and DBP 80 to 95 mmHg [172]. Patients were divided into 3 intervention groups: one-time advice, “established” behavioral intervention based on the established recommendations, and “established plus DASH.” Food was prepared by participants and they were followed up by telephone interview. The results showed that compared with the group receiving advice only, the 2 groups with behavioral interventions had significant decrease in the prevalence of hypertension and a higher percentage of optimal blood pressure at 6 month followup. However, there was no statistically significant difference between them.

### 11.2. Vegetarian Diet

Early observation has described lower blood pressure in vegetarians compared to patients on regular diets [173]. It seemed that sodium intake in vegetarian diet did not contribute a significant effect to blood pressure changes [174]. Recent studies also demonstrated that vegans do have lower SBP and DBP and less likely to use antihypertensive medications. In a study, for vegans, the odds ratio of hypertension compared with omnivores was 0.37 (95% CI, 0.19 to 0.74) [175]. Vegetarian diet with increased intake of fruit and vegetables, polyunsaturated vegetable margarines, and oils, fiber, calcium, and magnesium and decreased intake of protein in mild untreated hypertensive patients resulted in a fall of 5 mmHg in SBP. This diet improved blood pressure without a change in urinary sodium or potassium excretion or body weight [176]. Rouse and colleagues included 59 healthy normotensive patients in a randomized controlled study in which they were randomized to a control group (omnivorous diet) and one of 2 experimental groups (omnivorous and/or lacto-ovo-vegetarian diet). Mean SBP dropped by 5 to 6 mmHg and DBP dropped by 2 to 3 mmHg in the group on vegetarian diet after adjustment for age, obesity, heart rate, weight change, and blood pressure before dietary change. Blood pressure rose substantially in subjects who reverted to the omnivorous diet [177].

It appears that a vegetarian diet might reduce blood pressure by several factors: increased vegetables, fiber and fruit intake, low protein, and so forth.

## 12. Conclusion 

Dietary modification has important therapeutic roles in blood pressure control. Strong evidence supports the recommendation of a diet containing high potassium, moderate alcohol, and high fiber intake. As a whole, a DASH pattern diet rich in fruits, vegetables, low-fat dairy products, whole grains, nuts, and fish with reduced amount of red meat, fat, sugar-sweetened food and beverages, and/or a vegetarian diet, which is also high in vegetables and fruits and low in animal protein should be considered. The use of pharmacological supplements to achieve these dietary goals is not advised. Sodium restriction is strongly recommended by professional organizations, however, this needs to be carefully interpreted and individualized given the evidence of associated potential harmful effects and its unequal efficacy in various patient populations. The recommendation is not quite definite in terms of increased intake of calcium, magnesium, fish oil, and garlic. Irregular coffee drinkers and licorice consumers face a possibility of inducing hypertension; thus, these habits need to be avoided in patients at risk. 

Established nutrition recommendations are proven to be helpful in reducing blood pressure and thus hypertension-related complications and overall mortality. However, in view of the heterogeneity in risk factors, patient features, and pathogenesis of hypertension, the approach should be individualized and discussed in detail between the patient and their physician and/or dietician, based on the understanding of each patient's distinct disease characteristics.

The importance of dietary modification in hypertension management has been substantially studied for past decades with encouraging findings. Additional studies are warranted to further explore the role of these nutrient factors in preventing and treating this condition in special populations. 
